# Preserved Myocardial Deformation after Successful Coarctation Repair: A CMR Feature-Tracking Study

**DOI:** 10.1007/s00246-017-1788-1

**Published:** 2017-12-05

**Authors:** Elles J. Dijkema, Martijn G. Slieker, Johannes M. P. J. Breur, Tim Leiner, Heynric B. Grotenhuis

**Affiliations:** 1 Department of Pediatric Cardiology, Wilhelmina Children’s Hospital, P.O. Box 85090, 3508 AB Utrecht, The Netherlands; 2 Department of Pediatric Cardiology, Radboud University Medical Center, Nijmegen, The Netherlands; 3 Department of Radiology, University Hospital Utrecht, Utrecht, The Netherlands

**Keywords:** Coarctation of the aorta, Congenital heart disease, Feature tracking, Myocardial deformation

## Abstract

Arterial vasculopathy and residual aortic obstruction can lead to left ventricular (LV) dysfunction in patients with coarctation of the aorta (CoA) related to adverse ventriculo-arterial coupling. This study aimed to investigate potential differences in LV myocardial deformation indices between repaired CoA patients and healthy controls. Twenty-two CoA patients (age 30 ± 10.6 years) after surgical repair (*n* = 12) or balloon angioplasty (BA) (*n* = 10) without residual stenosis, between 3 months and 16 years of age with > 10 years follow-up were compared to 22 healthy age- and gender-matched controls (age 30 ± 3.8 years). Cardiac magnetic resonance feature tracking (CMR-FT) was used for LV longitudinal-, circumferential-, and rotational deformation indices. Global systolic LV function was preserved in CoA patients (LV ejection fraction 58 ± 4.8 vs. 60 ± 6.8%, *p* = 0.56) when compared to controls, with normal LV dimensions and mass (*p* > 0.05). Twelve CoA patients (55%) were hypertensive, of whom 4 were on anti-hypertensive medication. LV global longitudinal strain was preserved in the four-chamber (− 18 ± 4.4 vs. − 16 ± 4.7%, *p* = 0.06) and two-chamber (− 22 ± 5.1 vs. − 20 ± 6.0%, *p* = 0.22) orientations in CoA patients. Global circumferential strain was preserved at basal (− 29 ± 4.1 vs. − 28 ± 4.8%, *p* = 0.43), mid-ventricular (− 27 ± 4.2 vs. − 25 ± 3.0%, *p* = 0.09), and apical levels (− 35 ± 7.8 vs. − 32 ± 34.9%, *p* = 0.32). No differences were found in global torsion (2.4 ± 1.3° vs. 2.0 ± 1.4°/cm, *p* = 0.28), twist (14 ± 5.8° vs. 12 ± 6.3°, *p* = 0.34), and recoil rate (− 17 ± 9.7° vs. − 17 ± 7.1°/cm s, *p* = 0.97). Analysis of intra-observer variability demonstrated good reproducibility for all CMR deformation indices. Global and rotational myocardial deformation indices are preserved in CoA patients long-term after repair without residual stenosis, despite a high incidence of hypertension.

## Background

Coarctation of the aorta (CoA) is defined as a localized narrowing of the aorta, which can be repaired through endovascular and/or surgical treatment [1, 2]. Despite adequate relief of the stenosis, CoA patients still have a reduced life expectancy and an increased risk for cardiovascular complications including hypertension, left ventricular (LV) dysfunction, atherosclerosis, and coronary artery- and cerebrovascular disease [3]. These long-term increased risks for morbidity and mortality suggest residual and/or progressive cardiovascular alterations long-term after CoA repair [3, 4]. Coarctation is associated with aortic vasculopathy, which may lead to increased stiffness of the arterial vascular bed [4]. Residual aortic obstruction in CoA patients is also associated with increased LV afterload and adverse ventricular–arterial coupling, ultimately resulting in LV myocardial dysfunction [5–10]. Whether adverse ventricular–arterial coupling is also present in well-repaired CoA patients is unclear.

Previous studies have demonstrated preserved or even increased global cardiac function, e.g., LV ejection fraction (LVEF), in patients treated for CoA [6, 11]. However, LVEF has been demonstrated to be a poor determinant of cardiovascular events as LVEF can remain within normal range despite ventricular dysfunction [11, 12]. Recent reports indicate that quantification of myocardial deformation provides a better insight in global and regional ventricular function, with a strong correlation to cardiovascular outcome [13–15]. Cardiovascular magnetic resonance imaging (CMR) is currently the preferred advanced imaging modality for non-invasive follow-up of congenital heart disease [2, 16]. Recently developed CMR feature-tracking (CMR-FT) software provides fast and accessible analysis of myocardial deformation of standard CMR cine images [17–19]. This study therefore aimed to investigate CMR-FT myocardial deformation indices of the LV in CoA patients long-term after repair without residual stenosis, with comparison between two treatment strategies: surgical repair and balloon angioplasty (BA).

## Methods

### Study Population

The study population consisted of 22 patients who were successfully treated for CoA (no signs of obstruction on echocardiographic imaging, arm–leg blood pressure gradient < 20 mmHg) with surgery or balloon angioplasty (BA) and had a follow-up of at least 10 years. Patients who underwent primary repair of localized membranous CoA between 3 months and 16 years of age between 1969 and 2004 in our institution were included in the study. To allow for comparison between surgery and BA, CoA patients with primary repair before 3 months of age were not included as surgical repair was routine for those patients. Patients with isthmus hypoplasia, aortic arch hypoplasia, and/or severe associated heart defects (e.g., hypoplastic left heart syndrome, transposition of the great arteries) were excluded. Twenty-two of the 72 included patients agreed to undergo CMR testing (inclusion rate: 31%). Controls were recruited from a local cohort of healthy volunteers and were age- and gender matched to the CoA patient group.

### Imaging

CMR imaging was performed on a 1.5 T scanner (Ingenia R4.1.2; Philips Healthcare, Best, The Netherlands). Cine images were obtained using a electrocardiogram-gated breath-hold balanced fast-field echo sequence (SSFP) in long-axis planes (two- and four-chamber views) and short-axis slices covering the LV from the annulus of the mitral valve to the LV apex. Results for cardiac dimensions and LVEF were derived from short-axis slices according to the Society for Cardiovascular Magnetic Resonance recommendations [20]. End-diastolic LV mass, LV end-diastolic volume (EDV), and LV end-systolic volume (ESV) were indexed to body surface area (BSA) as described by Mosteller [21].

### CMR Analysis

Cardiac magnetic resonance feature-tracking analysis was performed using feature-tracking software (2D Cardiac Performance Analysis MR 1.0; TomTec Imaging Systems, Unterschleissheim, Germany), based on tissue voxel motion tracking to analyze mechanical deformation [22]. Initial endo- and epicardial borders are drawn manually in the longitudinal and short-axis images [22]. The software subsequently assigns several characteristics (i.e., brightness, inhomogeneities, tissue patterns, and anatomic structures) to 48 tissue voxels in the initially set contour and tracks these voxels frame-by-frame using a hierarchical algorithm [22]. Movement of the features throughout the cardiac cycle enables derivation of deformation parameters such as strain and strain rate, as well as myocardial velocity and rotation [22]. Endocardial and epicardial borders were manually drawn in end-diastole with exclusion of papillary muscles in the endocardial border. The automatically propagated contour was assessed visually and adjustments were made manually in case of inappropriate tracking.

**Fig. 1:**
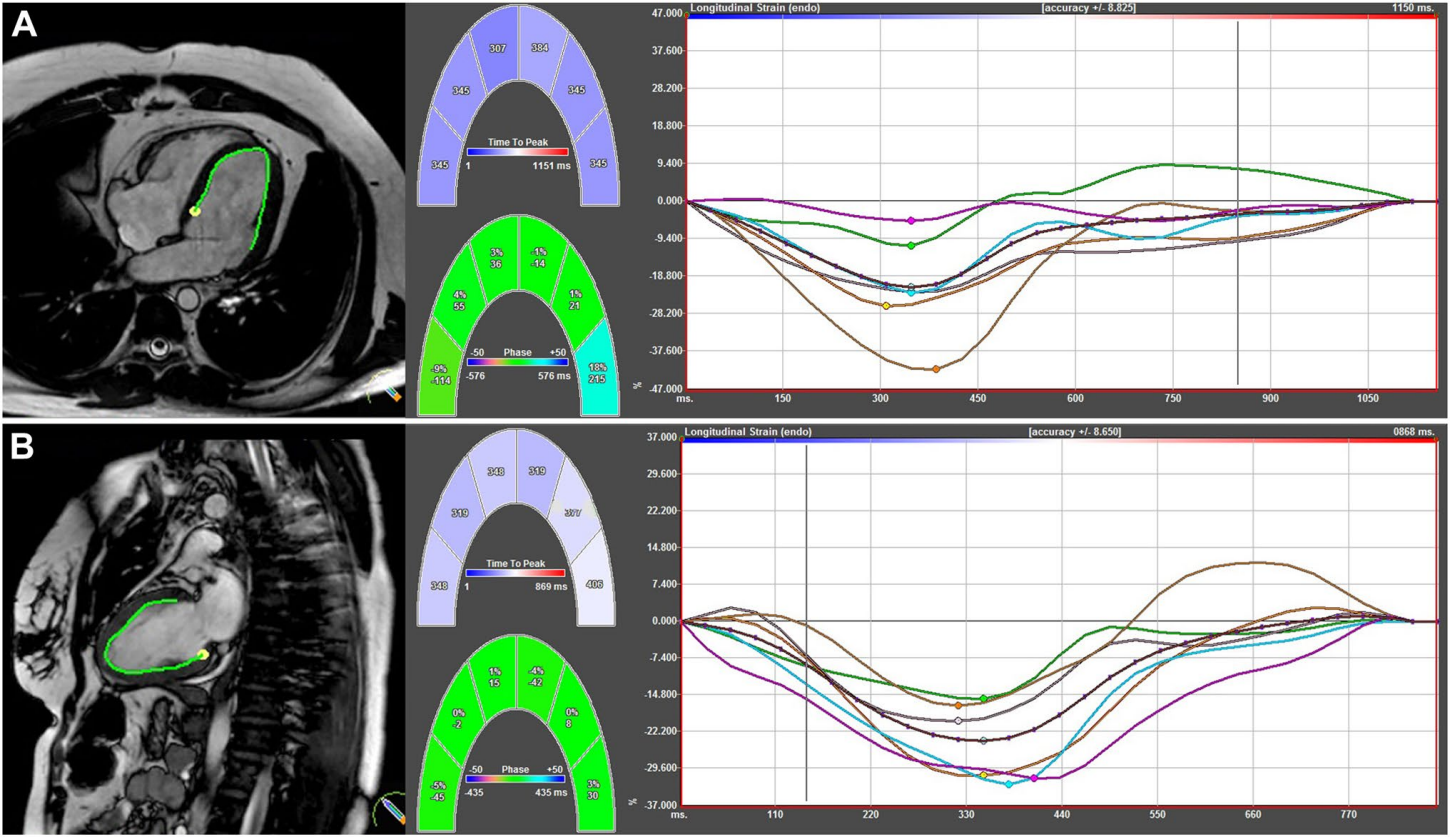
Longitudinal myocardial strain analysis using CMR Feature tracking in **a** 4-chamber and **b** 2-chamber view. The left panels show endocardial tracking in 4- and 2-chamber views, resulting in horizontal (top) and vertical (bottom) strain curves. The coordinated curves with synchronized peak strain indicate preserved myocardial deformation

**Fig. 2:**
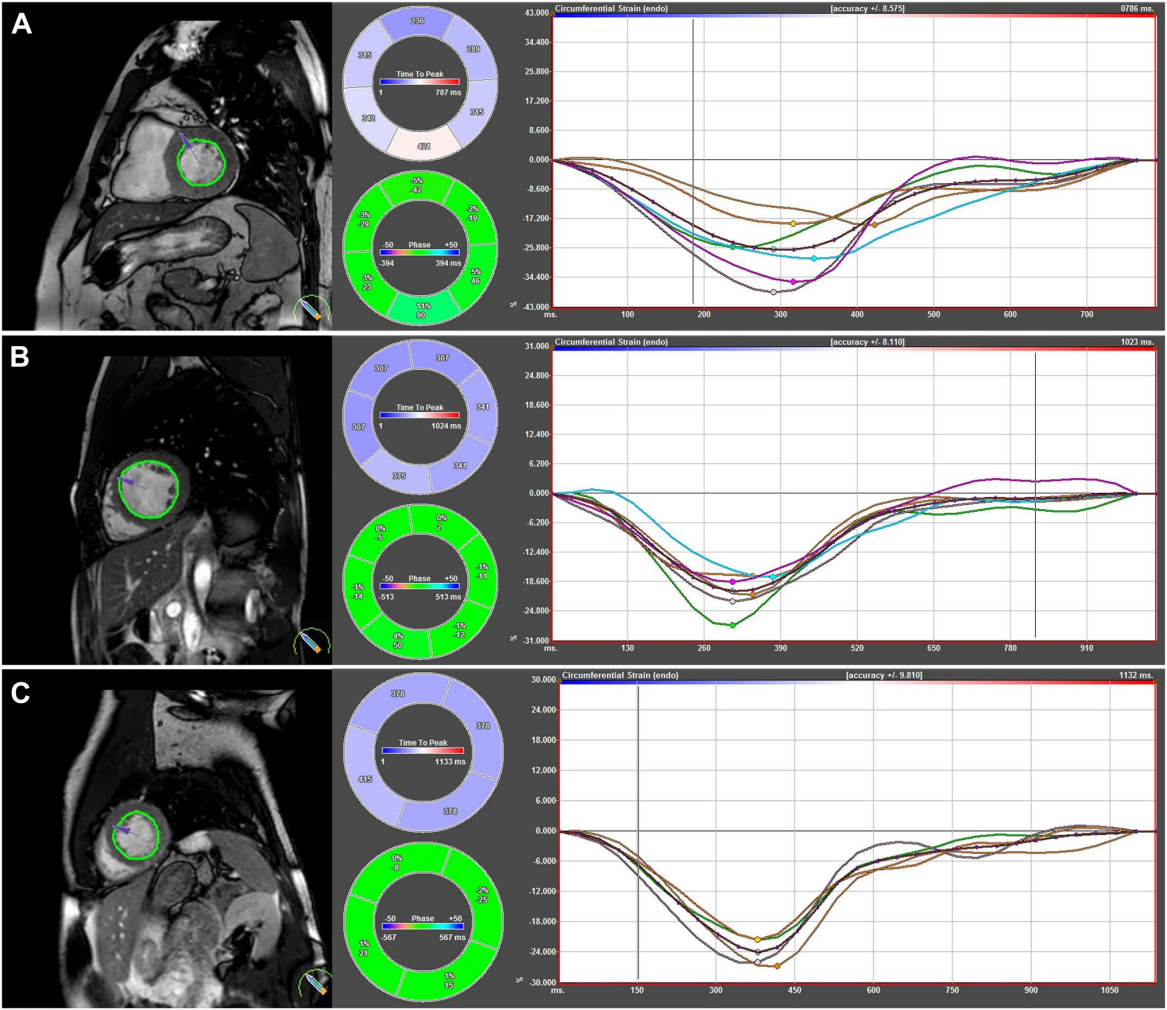
Circumferential myocardial strain analysis using CMR Feature tracking in **a** basal, **b** mid-ventricular, and **c** apical sagittal view. The left panels show endocardial tracking in basal- (top), mid-ventricular- (middle), and apical (bottom) views. The coordinaded curves with synchronized peak strain indicate preserved myocardial deformation

Longitudinal strain, strain rate, velocity, and displacement were measured in two- and four-chamber long-axis views (Fig. 1). Circumferential strain, strain rate, velocity, and displacement, as well as rotation and rotation rates were measured in short-axis views at basal (slice closest to the base with a full circle of myocardium), mid-ventricular (slice where the papillary muscles are free from the wall), and apical levels (apical slice showing LV cavity at end-systole, around 25% LV distance, as this is has been shown to be the most appropriate apical location for analysis of rotational deformation [23]) (Fig. 2).

Myocardial deformation was analyzed in 16 myocardial segments, in accordance with the American Heart Association’s standardized myocardial segmentation [10]. If adequate tracking of certain segments was not possible, these segments were excluded from analysis. Inadequate tracking was defined as a tracking deviation from the moving myocardial border > 50% of the myocardial width [18]. All myocardial deformation indices were measured three times in each patient in all views, and an average of all three measurements was used for results. Global longitudinal and circumferential strain was calculated as the average of the peak strain values of the subsequent segments.

Rotation was calculated in systole and diastole for the basal and apical slices, by averaging the rotation for all segments in the respective slice relative to the LV center axis. Positive rotation is considered counterclockwise when viewed from caudal to cranial position [22]. Torsion was calculated as the difference in apical (counterclockwise) and basal (clockwise) rotation divided by the inter-slice distance [23]. Calculation of torsion allows for comparison of twist deformation between patients with different cardiac sizes, especially applicable to the pediatric population [22]. Peak recoil rate was calculated as the maximum slope of the torsion–time curve as a measure of LV diastolic performance [22].

### Statistical Analysis

Data are expressed as mean ± standard deviation. Statistical comparisons of longitudinal-, and circumferential strain and strain rates, torsion and recoil rates between the CoA group, subgroups and normal controls were performed using unpaired Student’s *t* tests and one-way analysis of variance. Multiple regression was used to assess correlations between different cardiac variables and deformation indices. Intra-observer variability was assessed using the intraclass correlation coefficient (ICC). Data analyses were performed using SPSS, version 22 (International Business Machines Corporation, Armonk, New York, United States). A *p* value < 0.05 was considered statistically significant.

## Results

The study population consisted of 22 CoA patients and 22 healthy controls. Baseline characteristics of the study population are presented in Table 1. All CoA patients were primarily treated with surgery by end-to-end anastomosis (12 patients) or BA (10 patients). Mean age at intervention was 5.9 years (range 0.35–14.4 years) and mean follow-up at the time of CMR was 23.9 years (range 10.8–42.4 years). Surgical patients were significantly older than BA patients (35 ± 9.5 vs. 22 ± 6.7 years, *p* < 0.01), with a longer follow-up to CMR (29 ± 8.5 vs. 18 ± 2.9 years, *p* < 0.01), as BA is a more recently introduced technique in our practice. Systolic-, diastolic-, and mean arterial blood pressures (SBP, DBP, MAP) were 135 ± 13.2, 76   ±  8.5, and 96 ± 8.8 mmHg, respectively, for CoA patients. Twelve CoA patients (55%) were hypertensive according to the European Society of Hypertension Guidelines (SBP ≥ 95th percentile) [24]) at the time of investigation, of whom seven (58%) had undergone surgery and five (42%) BA. Five patients were using anti-hypertensive medication, of whom four were still hypertensive at the time of investigation. None of the normotensive CoA patients were on anti-hypertensive medication. Nine patients (41%) had a bicuspid aortic valve, none of which had significant valve dysfunction. Five patients (23%), two BA and three surgical patients, had been successfully treated for re-CoA: two underwent surgery, one received BA, and two were treated with an endovascular stent. The median time between primary repair and re-CoA was 8.8 years (mean 13.8 years; range 2.1–40.4 years). LV mass showed a significant positive correlation with age at time of repair (*R* ^2^ = 0.78, *p* = 0.046). No significant correlation was found between LV mass and age, blood pressures, time between repair and CMR, the repair to reintervention interval, or the reintervention to CMR interval (*R* ^2^ = 0.01–0.66, *p* > 0.05). Systolic blood pressure was not significantly correlated with age, BSA, LV mass, intervention to CMR interval, age at time of repair, repair to reintervention interval, or reintervention to CMR interval (*R* ^2^ = 0.03–0.69, *p* > 0.05).

**Table 1:** Baseline characteristics of the study population

	CoA	Control	*p* value
	*n* = 22	*n* = 22	
Age	30 ± 10.6	30 ± 3.8	0.87
BSA	1.9 ± 0.22	2.0 ± 0.12	0.81
MAP	96 ± 8.8	94.8 ± 8.5	0.72
SBPday	135 ± 13.2	129 ± 10.8	0.12
HR	65 ± 11.0	60 ± 9.7	0.11
Intervention			
Surgery	12 (55%)	–	
BA	10 (45%)	–	
Hypertension	12 (55%)	–	
BAV	9 (41%)	–	
Reintervention	5 (23%)	–	

*CoA* coarctation of the aorta, *BSA* body surface area, *MAP* mean arterial pressure, *SBPday* systolic blood pressure during daytime, *HR* heart rate, *BA* balloon angioplasty, *BAV* bicuspid aortic valve

### Global Cardiac Function

All CoA patients had preserved global systolic LV function when compared to controls (LVEF 59 ± 4.8vs. 60 ± 6.8%, *p* = 0.56) (Table 2). LV mass, LVEDV, and LVESV were also normal in CoA patients (*p* ≥ 0.05 for all). No significant difference in LVEF was found between patients treated for re-CoA and patients without re-CoA (LVEF 59 ± 3.9vs. 59 ± 5.1%, *p* = 0.75).

**Table 2:** Global left ventricular (LV) function and LV deformation in coarctation versus healthy controls

	CoA	Controls	*p* value
	*n* = 22	*n* = 22	
LVEF	59 ± 4.77	60 ± 6.84	0.56
LV mass	68 ± 8.49	64 ± 8.63	0.08
EDV	93 ± 15.2	94 ± 9.3	0.79
ESV	26 ± 7.0	31 ± 7.3	0.05
Strain			
GLS			
Horizontal	− 18.5 ± 4.37	− 15.8 ± 4.69	0.06
Vertical	− 21.8 ± 5.10	− 19.7 ± 6.04	0.22
GCS			
Basal	− 29.3 ± 4.06	− 28.2 ± 4.79	0.43
Mv	− 27.0 ± 4.19	− 25.1 ± 2.96	0.09
Apex	− 34.6 ± 7.79	− 32.6 ± 4.88	0.32
Strain rate			
GLSR			
Horizontal	− 0.8 ± 0.23	− 0.7 ± 0.22	0.02
Vertical	− 1.1 ± 0.31	− 0.8 ± 0.30	0.02
GCSR			
Base	− 1.5 ± 0.30	− 1.4 ± 0.31	0.25
Mv	− 1.4 ± 0.31	− 1.3 ± 0.25	0.33
Apex	− 1.9 ± 0.47	− 1.7 ± 0.32	0.07
Rotation			
Twist	12.3 ± 6.29	14.2 ± 5.83	0.33
Torsion	2.0 ± 1.35	2.4 ± 1.35	0.28
Recoil rate	− 17.8 ± 7.11	− 17.7 ± 9.73	0.97

LV mass, LVEDV, and LVESV are indexed to body surface area

*CoA* coarctation of the aorta, *LVEF LV* ejection fraction, *LVEDV* end-diastolic volume, *LVESV* end-systolic volume, *MAP* mean arterial pressure, *SBPday* mean systolic blood pressure during daytime, *HR* heart rate, *GLS* global longitudinal strain, *GCS* global circumferential strain, *GLSR* global longitudinal strain rate, GCSR global circumferential strain rate

### Left Ventricular Myocardial Deformation

LV global longitudinal strain in the four-chamber and two-chamber planes was similar between CoA patients and controls (*p* = 0.06 and *p* = 0.22, respectively). Global circumferential strain was also preserved at basal (*p* = 0.43), mid-ventricular (*p* = 0.09), and apical levels (*p* = 0.32). Global longitudinal strain rate was increased in CoA patients when compared to controls (four-chamber: − 0.8 ± 0.23 vs. − 0.7 ± 0.22%, *p* < 0.05; two-chamber: − 1.1 ± 0.31 vs. − 0.8 ± 0.30%, *p* < 0.05). Global torsion (*p* = 0.28), twist (*p* = 0.34), and recoil rate (*p* = 0.97) demonstrated no differences between CoA patients and controls (*p* = 0.28, 0.34 and 0.97, respectively).

Global longitudinal strain was significantly correlated with LVEF (*R* ^2^ = 0.234, *p* < 0.01), and global circumferential strain was significantly correlated with LVEF at the mid-ventricular and apical levels (*R* ^2^ = 0.01, *p* = 0.01 and *R* ^2^ = 0.09, *p* = 0.047, respectively). Basal circumferential strain showed a significant correlation with age at the time of repair (*R* ^2^ = 0.29, *p* < 0.01). None of the studied LV myocardial deformation indices correlated with SBP, DPB, and MAP (*R* ^2^ = 0.001–0.156, *p* > 0.05 for all indices).

### Balloon Angioplasty Compared to Surgery

Patients treated with BA were significantly younger at time of investigation (22 ± 6.7 vs. 36 ± 9.5 years, *p* < 0.01) and showed a slightly higher LVEF (61 ± 4.0 vs. 57 ± 4.7%, *p* < 0.05) when compared to CoA patients treated with surgery. Two surgical patients had an LVEF below 55% (48 and 49%, respectively), versus one BA patient (54%). No CoA patient had an LVEF < 40%. Global strain and rotational indices between the two patient groups were comparable (*p* > 0.05), despite a slightly decreased apical circumferential strain rate in surgical CoA patients (− 1.7 ± 0.47 vs. − 2.1 ± 0.38/s, *p* < 0.05). No significant differences in SBP (136 ± 13 vs. 133 ± 14 mmHg, *p* = 0.66), DBP (75 ± 4 vs. 78 ± 12 mmHg, *p* = 0.56), and MAP (96 ± 6 vs. 96 ± 12 mmHg, *p* = 0.88) were found between surgical and BA patients.

### Hypertension

Hypertensive CoA patients (with SBP ≥ 95th percentile according to the European Society of Hypertension Guideline [24]) demonstrated a similar LV mass when compared to normotensive CoA patients or controls (68 ± 8.9 vs. 67 ± 8.4 g/m^2^ and 64 ± 8.6 g/m^2^, *p* = 0.20) and a similar LV mass to LVEDV ratio (0.83 ± 0.14 vs. 0.90 ± 0.18 g/ml and 0.85 ± 0.15 g/ml, *p* = 0.59). No significant differences were found for LVEF (*p* = 0.83) or LV dimensions (*p* = 0.62 and 0.09 for LVEDV and LVESV, respectively). Hypertensive CoA patients demonstrated a slightly increased global longitudinal strain in the four-chamber plane when compared to normotensive patients and controls (− 19.4 ± 3.33 vs. − 17.4 ± 5.34% and − 15.8 ± 4.69%, respectively, *p* = 0.10), but this difference was not statistically significant (Table 3). No significant differences were found in strain rate or rotational indices between hypertensive CoA patients, normotensive patients, and controls.

**Table 3:** Global left ventricular (LV) function and LV deformation in normotensive- and hypertensive CoA patients and controls

	Controls	CoA NT	CoA HT	*p*
	*n* = 22	*n* = 10	*n* = 12	
LVEF	60 ± 6.8	59 ± 6.3	58 ± 3.3	0.83
LV mass	63 ± 8.6	67 ± 8.4	68 ± 8.9	0.21
EDV	94 ± 8.6	90 ± 13.3	96 ± 16.7	0.62
ESV	31 ± 7.3	24 ± 6.9	27 ± 7.1	0.09
MAP	94.8 ± 8.5	89 ± 4.2	101 ± 8.0	< 0.01
SBPday	129 ± 10.8	123 ± 6.4	144 ± 9.3	< 0.01
HR	60 ± 9.7	62 ± 10.8	68 ± 10.8	0.12
Strain				
GLS				
Horizontal	− 15.8 ± 4.69	− 17.4 ± 5.34	− 19.4 ± 3.33	0.10
Vertical	− 19.7 ± 6.04	− 22.8 ± 5.06	− 21.1 ± 5.24	0.38
GCS				
Basal	− 28.2 ± 4.79	− 29.7 ± 4.97	− 28.9 ± 3.31	0.68
Mv	− 25.1 ± 2.96	− 27.7 ± 2.62	− 26.4 ± 5.19	0.17
Apex	− 32.6 ± 4.88	− 35.1 ± 8.01	− 34.2 ± 7.93	0.58
Strain rate				
Glsr				
Horizontal	− 0.7 ± 0.22	− 0.8 ± 0.25	− 0.8 ± 0.21	0.07
Vertical	− 0.8 ± 0.30	− 1.1 ± 0.31	− 1.0 ± 0.33	0.07
GCSR				
Base	− 1.4 ± 0.31	− 1.5 ± 0.31	− 1.5 ± 0.32	0.51
Mv	− 1.3 ± 0.25	− 1.4 ± 0.21	− 1.4 ± 0.37	0.48
Apex	− 1.7 ± 0.32	− 1.9 ± 0.49	− 1.9 ± 0.48	0.17
Rotation				
Twist	12.3 ± 6.29	14.9 ± 5.92	13.6 ± 5.95	0.55
Torsion	2.0 ± 1.35	2.7 ± 1.64	2.2 ± 1.07	0.34
Recoil rate	− 17.8 ± 7.11	− 19.7 ± 12.71	− 16.0 ± 6.45	0.60

LV mass, LVEDV and LVESV are indexed to body surface area

*CoA* coarctation, *HT* hypertensive patients, *NT* normotensive patients, *Co* controls, *LVEF* LV ejection fraction, *EDV LV* end-diastolic volume, *ESV LV* end-systolic volume, *MAP* mean arterial pressure, *SBPday* mean systolic blood pressure during daytime, *HR* heart rate, *GLS* global longitudinal strain, *GCS* global circumferential strain, *GLSR* global longitudinal strain rate, *GCSR* global circumferential strain rate

*p* values are given in the three most right columns

### Reproducibility

CMR-FT demonstrated good intra-observer reproducibility, with an intraclass correlation coefficient > 0.8 for all global strain, strain rate, and rotational indices. All ICCs are presented in Table 4.

**Table 4:** The intraclass correlation coefficients (ICC) for global myocardial deformation measurements

Strain		ICC	95% CI
GLS	Horizontal	0.939	(0.875–0.973)
	Vertical	0.927	(0.850–0.968)
GCS	Basal	0.952	(0.903–0.979)
	Mid-ventricular	0.945	(0.890–0.976)
	Apical	0.980	(0.958–0.991)
Strain rate			
GLSR	Horizontal	0.859	(0.716–0.937)
	Vertical	0.925	(0.846–0.967)
GCSR	Basal	0.964	(0.928–0.984)
	Mid-ventricular	0.985	(0.970–0.993)
	Apical	0.938	(0.875–0.972)

*GLS* global longitudinal strain, *GCS* global circumferential strain, *GLSR* global longitudinal strain rate, *GCSR* global circumferential strain rate

## Discussion

Repaired CoA patients demonstrate an increased susceptibility for cardiovascular morbidity and mortality at long-term follow-up [9, 10, 25–27]. Residual aortic obstruction in CoA patients is associated with adverse ventricular–arterial coupling related to increased LV afterload [10]. Whether a similar cascade is also present in well-repaired CoA patients is unknown. The present study quantifies myocardial deformation in CoA patients long-term after surgical or BA repair without residual stenosis. This study adds the following to our understanding of cardiovascular health in well-repaired CoA patients long-term after repair:

- global LV systolic function is generally normal;
- global and regional myocardial deformation indices are preserved in well-repaired CoA patients, despite a high incidence of hypertension;
- the choice for treatment modality—surgery versus BA—has no impact on LV systolic function or LV myocardial deformation.

### Global Cardiac Function

Global LV function was preserved in our cohort of well-repaired CoA patients at long-term follow-up as previously described, expressed by normal LVEF [4, 9, 12, 26]. Our CoA cohort also demonstrated normal LV mass and LV dimensions, suggesting that no significant LV remodeling related to potentially increased LV afterload is present in well-repaired CoA patients, which is consistent with other CMR and echocardiographic reports [9, 10, 26]. In contrast, several echocardiographic studies showed increased LV mass after CoA repair, although these studies included CoA patients treated at significantly older age or with a longer follow-up duration (> 20 years) [4, 11, 25]. Increased LV mass is thought to be the result of increased afterload and has been related to residual aortic stenosis at the site of previous repair [26]. Our study demonstrates that repaired CoA patients without residual stenosis not only have preserved LV systolic function by LVEF, but also normal myocardial mass.

### Left Ventricular Myocardial Deformation

Myocardial deformation was preserved in our CoA patients when compared to healthy controls, in agreement with previous echocardiography and CMR-FT reports indicating preserved longitudinal and circumferential strain in CoA patients with preserved global LV systolic function and normal LV mass [9, 11]. Jashari et al. found reduced LV global longitudinal strain and strain rate prior to surgical CoA repair, with a further decrease shortly after intervention [28]. Interestingly, progressive normalization of strain was observed over 2 years after satisfactory repair, resulting in near-normal LV longitudinal strain values [28]. Kowalski et al. also showed normal global LV function and LV longitudinal strain in CoA patients by 2D echocardiographic deformation 10 years after surgical repair, suggesting a full recovery of the myocardium at long-term follow-up [11]. In contrast, a recent CMR-FT study by Shang et al. showed impaired longitudinal deformation in CoA patients treated in the neonatal period [10]. This study included well-repaired CoA patients treated with surgery, BA, or stent placement at an early age (before 3 months of age) [10]. In the current study, patients treated before 3 months of age were excluded, as most patients treated at this young age suffer from a more severe coarctation, requiring immediate correction. Abnormal longitudinal deformation was explained by Shang et al. by increased arterial stiffness and altered ventriculo–arterial coupling [10]. The pulse wave velocity in the proximal aorta was increased with increased aortic stiffness, resulting in an early return of the reflected wave, imposing increased afterload and impaired LV relaxation [10].

Surprisingly, our CoA patients demonstrated increased systolic global longitudinal strain rate when compared to our control group. Systolic strain rate has been identified as a strong indicator for LV contractility. Increased contractile performance has been described in CoA patients after repair, as CoA patients experience increased afterload which may lead to myocardial remodeling and LV hypertrophy to maintain normal LV wall stress levels [29]. Kimball et al. suggested that repair of coarctation may relieve the increased afterload, but myocardial hypertrophy may partially persist which results in a hypercontractile myocardial state after repair. In addition, GLS demonstrated a trend towards lower GLS in controls, which may reflect the same phenomenon of a hypercontractile state in the CoA patient group.

Conflicting data on myocardial deformation after CoA repair may be explained by differences in age at the time of repair, associated severity of CoA, and duration of follow-up between studies. The myocardial response to increased pressure load is age-dependent as the myocardium has the ability to regenerate and increase vascularity in children, thereby restoring pressure-induced remodeling with preservation of coronary blood flow and reduction of development of myocardial fibrosis [12, 30]. Delayed repair of CoA may induce a maladaptive response of the LV, resulting in adverse outcome later in life [12]. Age at the time of repair has therefore been identified as a predictor for impaired longitudinal strain [31]. In addition, the set point of the renin–angiotensin–aldosterone system is defined in neonatal life [32]. Delayed CoA repair may therefore lead to more abnormal development of the renin–angiotensin–aldosterone system, which may in turn lead to hypertension and concomitant increased LV afterload [33, 34]. This study showed a significant correlation between age at time of repair and LV mass. However, we found no influence of age at time of repair on longitudinal strain in patients with mild-to-moderate CoA treated at a mean age of 5.9 years. Preserved longitudinal strain might suggest limited effects of adverse arterial–ventricular coupling in this patient group long-term after successful CoA repair. However, as assessment of aortic vascular function was not performed in this study, we can not assess the influence of arterial stiffness.

Jashari et al. reported more impaired longitudinal strain in CoA patients with LV hypertrophy, as confirmed by other studies [9, 27, 28, 31]. The myofiber orientation changes throughout the myocardium, with a predominantly longitudinal orientation in the endomyocardium [10, 27]. The endomyocardial fibers are most sensitive to pressure load-induced fibrosis [27]. Therefore, longitudinal strain is primarily affected in patients with pressure load-induced LV hypertrophy and LV myocardial dysfunction [27]. Well-repaired CoA patients in our cohort showed normal LV mass—hence the absence of compensatory LV hypertrophy—which may explain the preserved LV deformation indices. Our findings of preserved myocardial deformation after coarctation repair in the absence of LV hypertrophy are in line with a previous report of Kutty et al. [9], who demonstrated reduced longitudinal LV strain in CoA patients with increased LV mass and preserved longitudinal strain in patients with normal LV mass by use of CMR-FT [9]. In contrast to the study by Kutty et al. our current study also assessed the influence of hypertension on myocardial deformation. Our results demonstrate preserved myocardial deformation even in hypertensive patients in absence of LV hypertrophy, which might suggest that LV myocardial dysfunction occurs at a later stage in the cascade of arterial–ventricular coupling.

No difference in myocardial deformation was observed between CoA patients after BA and surgery in our study. Patients after surgery had slightly lower LVEF compared to BA patients, possibly due to the older age of the surgical patients. Similar results have been described by Chui et al. who showed a minor—non-significant—difference in LVEF between both treatment groups [35].

### Hypertension

Hypertension is a well-described long-term complication of CoA [3, 22, 36]. Hypertension is associated with an increased afterload, LV hypertrophy, and myocardial fibrosis [27]. Despite a high prevalence of hypertension in our patient group, LV mass and LV dimensions were preserved in our CoA patients. Hypertension did not affect global LV function or myocardial deformation in our cohort, suggesting that hypertension does not significantly affect cardiac function of well-repaired CoA patients at this point in follow-up. In fact, hypertensive CoA patients seemed to have slightly increased strain compared to controls. This difference is most likely not clinically relevant, as both strain values fall within normal range [17]. Left ventricular remodeling and hypertrophy with impaired LV deformation have been described in hypertensive patients [37–41]. In contrast to our current findings, Shang et al. reported increased LV mass and decreased myocardial deformation in hypertensive CoA patients compared to healthy controls. However, in accordance to our results, LV mass and myocardial deformation did not differ significantly between normotensive and hypertensive CoA patients [10]. Indeed, increased systolic blood pressure has been associated with concentric hypertrophy (increased LV mass combined with increased relative wall thickness) [42]. However, development of myocardial concentric hypertrophy is a gradual progressive process, which is usually preceded by concentric remodeling in which the relative wall thickness is increased, but LV mass is preserved [42].

### Limitations

A few limitations should be addressed regarding this study. The relatively small sample size limits the generalizability of the results and may have obscured further differences and associations. Our CoA patients were not analyzed for arterial stiffness, so direct assessment of ventriculo–arterial coupling could not be performed. Nevertheless, this study provides insight in myocardial deformation in CoA patients at long-term follow-up, suggesting good LV function in well-repaired patients. The long-term implications of our findings need to be substantiated with follow-up studies.

## Conclusions

Feature tracking by CMR provides easily accessible and reproducible analysis of myocardial deformation. Well-repaired CoA patients show preserved cardiac function by normal LV ejection fraction and normal myocardial deformation indices at long-term follow-up, despite a high incidence of hypertension. We found good reproducibility for strain, strain rate, and rotational indices with CMR-FT, suggesting CMR-FT is a useful tool for analysis of myocardial deformation.
